# Complete Genome Sequence of Hepatitis E Virus Genotype 3 Obtained from a Chronically Infected Individual in Uruguay

**DOI:** 10.1128/MRA.00367-21

**Published:** 2021-06-03

**Authors:** Florencia Cancela, Yanina Panzera, Victoria Mainardi, Solange Gerona, Natalia Ramos, Ruben Pérez, Juan Arbiza, Santiago Mirazo

**Affiliations:** a Sección Virología, Facultad de Ciencias, Universidad de la República, Montevideo, Uruguay; b Sección Genética Evolutiva, Facultad de Ciencias, Universidad de la República, Montevideo, Uruguay; c Programa de Trasplante Hepático, Hospital Central de las Fuerzas Armadas, Montevideo, Uruguay; Portland State University

## Abstract

Hepatitis E virus (HEV) is a leading cause of acute viral hepatitis worldwide. We report the full-length genome sequence of an HEV-3 strain obtained from a chronically infected patient from Uruguay. This strain shared only 86% nucleotide sequence identity with the most closely related reference strain belonging to subtype 3m.

## ANNOUNCEMENT

Hepatitis E is a zoonotic infection of increasing concern and a leading cause of acute hepatitis of viral origin in developed regions and regions of nonendemicity (1). The etiological agent, hepatitis E virus (HEV), belongs to the *Orthohepevirus A* species, *Hepeviridae* family, and is classified into 8 genotypes (HEV-1 to HEV-8), of which HEV-1 to HEV-4 and HEV-7 are recognized as human pathogens (2). Due to the extensive genetic diversity, HEV genotypes are further divided into subtypes (2, 3). HEV usually causes an acute and self-limiting disease in immunocompetent individuals. However, novel aspects regarding HEV infection have been recently uncovered, including the possibility of the disease becoming chronic in immunocompromised individuals or solid organ transplant (SOT) recipients (4).

We recently reported a case study of an autochthonous chronic HEV infection in a SOT recipient who was further diagnosed with a posttransplant lymphoproliferative disorder (5). That HEV strain, named C1, was identified as belonging to HEV-3, and in this study, we report the full-length genome sequence.

RNA was extracted from a stool sample with a Quick-RNA miniprep kit (Zymo Research Corp., USA). Double-stranded cDNA (ds-cDNA) was generated using a Maxima H Minus ds-cDNA synthesis kit (Thermo Fisher Scientific, USA) with random primers. The ds-cDNA was further amplified by multiple displacement amplification (MDA) technology using a REPLI-g minikit (Qiagen, Germany), followed by purification and quantification using an AMPure XP device (Beckman Coulter, USA) and a Qubit fluorometer (Qubit DNA high-sensitivity [HS] assay kit), respectively.

A Nextera DNA flex library preparation kit (Illumina, USA) with dual indexing was used with 50 ng of ds-cDNA. Quality control was performed on a Fragment Analyzer 5200 system (Agilent Technologies, USA) by using the standard-sensitivity next-generation sequencing (NGS) analysis kit (Agilent Technologies, USA). The library was sequenced on an Illumina MiniSeq genomic platform at Facultad de Ciencias (Universidad de la República [UdelaR], Uruguay) using a midoutput reagent cartridge (300 cycles, 150-bp paired-end reads) following the standard Illumina protocols.

A total of 286,928 sequencing raw reads were demultiplexed automatically on the MiniSeq platform with the default settings. Adapter/quality trimming and filtering were performed with the BBDuk plugin (default settings), and the reads were then mapped to the HEV genome (GenBank accession number FJ998008) using the Geneious mapper (medium-low sensitivity) available in the Geneious Prime 2020.2.1 software. Annotation was done with SeqMan NGen 12.0 (DNASTAR, Madison, WI). Complete sequence alignment, with ClustalW, and phylogenetic tree reconstruction were performed with MEGA X software (6).

**FIG 1:**
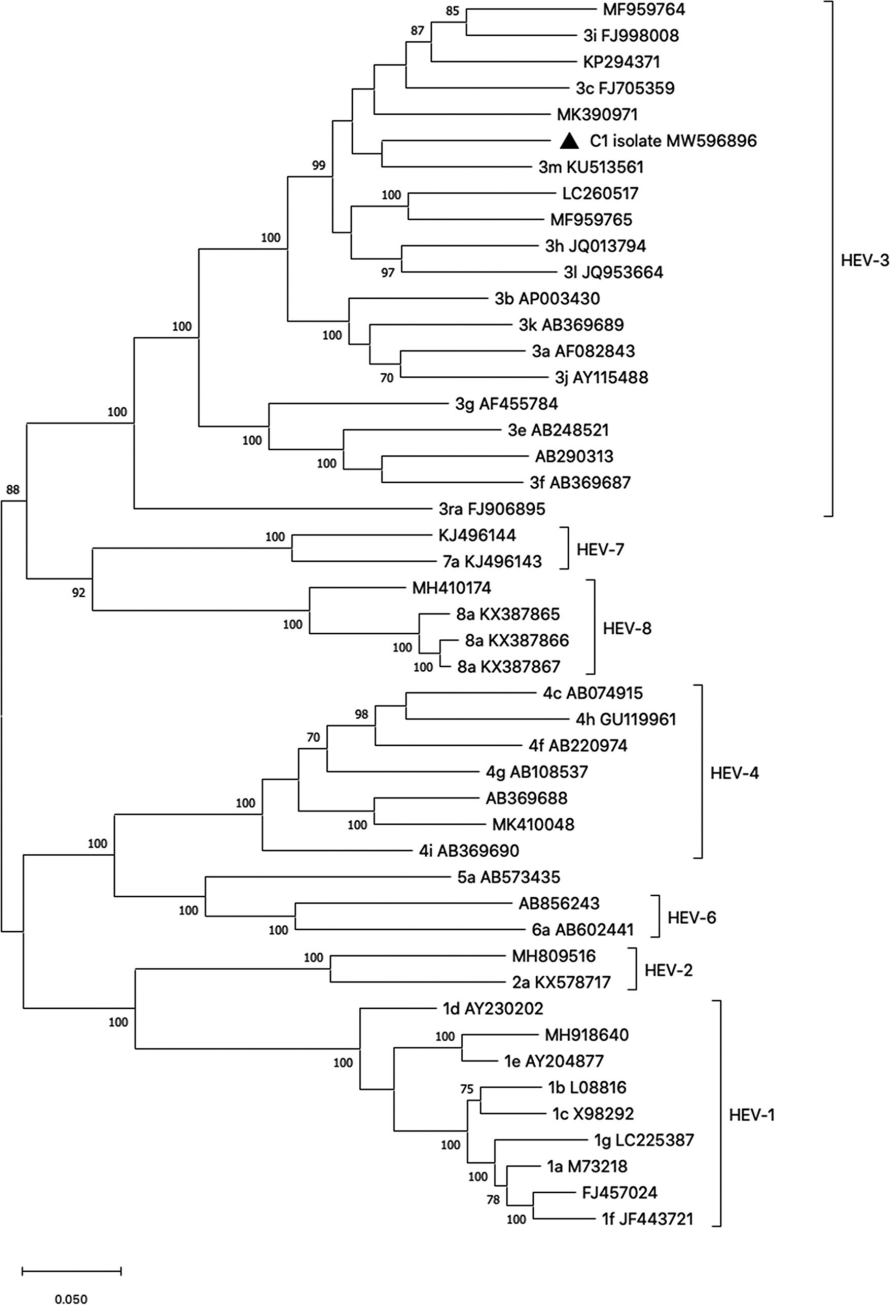
Phylogenetic tree based on the full-length genome sequences of proposed representative HEV genotype reference strains, according to Smith et al. (7). Subtype reference strains are also indicated. Tree reconstruction was performed using the maximum-likelihood method with Tamura-Nei as the best substitution model. The robustness of the tree was determined by bootstrap analysis (1,000 replicates), and only values of ≥60% are shown. The GenBank accession numbers are shown for each sequence. Letters indicate the subtype classification. The C1 strain from this study is marked with a solid triangle.

The complete sequence of the C1 strain was 7,229 nucleotides long with a 54.8% GC content, 7,507 mapped reads, and 114× coverage. The genome comprises three open reading frames (ORFs) that encode the viral proteins as follows: ORF1, positions 13 to 5124; ORF2, 5159 to 7141; and ORF3, 5121 to 5489. C1 clustered within HEV-3 sequences, and it shared 86% nucleotide sequence identity (complete sequence comparison in BLAST [https://blast.ncbi.nlm.nih.gov/Blast.cgi]) with the isolate KU513561, identified as the reference strain of the 3m subtype (7), the most closely related sequence according to the phylogenetic reconstruction (Fig. 1).

The use of the patient sample was approved by the Ethics Committee from the Hospital Central de las Fuerzas Armadas from Uruguay. The patient gave his written consent prior to the inclusion in this study.

To conclude, we present a complete HEV genome sequence obtained from a chronically infected patient. Further research is needed in order to attain deeper knowledge of the molecular epidemiology of HEV in Latin America.

### Data availability.

The complete genome sequence of C1 has been deposited in the GenBank database under the accession number MW596896 and in the SRA under BioSample accession number SAMN19016784 and study number PRJNA727355.
